# Pseudoduplication of the optic disc initially resembling a bifurcated optic nerve in a strabismus child: a case report

**DOI:** 10.1186/s12886-020-01369-1

**Published:** 2020-03-14

**Authors:** Liuhui Huang, Qi Zhang, Haiying Jin, Peiquan Zhao

**Affiliations:** 1 Department of Ophthalmology, Xinhua Hospital Affiliated to Shanghai Jiaotong University School of Medicine, Kongjiang Road, No. 1665, Shanghai, 200092 China; 2 Department of Ophthalmology, Tenth People’s Hospital Affiliated to Shanghai Tongji University School of Medicine, Shanghai, China

**Keywords:** Pseudoduplication, Double disc, Chorioretinal coloboma, Fluorescein angiography, Strabismus

## Abstract

**Background:**

Pseudoduplication of the optic disc is a rare clinical condition that is characterized by a circumscribed, disc-like lesion with radiating vessels but only one normal optic nerve. We report a rare case that initially resembled a bifurcated optic nerve in a strabismus child.

**Case presentation:**

A 6-year-old female child was initially referred to our hospital due to perceptual exotropia of 15 degrees with poor fixation of the left eye. The visual acuity of the left eye was 3/100 with a refraction of + 1.75/− 1.25 × 175. Fundus images of her left eye revealed a circumscribed and disc-like lesion located one disc diameter (DD) below the true optic disc that showed profound central cupping resembling a second optic disc with a vascular supply. B scan ultrasonography showed an optic nerve with a bifurcated weak-echo region, suggesting that two strands originated from the optic nerve. Optic coherence tomography (OCT) demonstrated a large crater-like depression of the lesion, indicating a colobomatous defect covered by a mysterious membranous structure, a disturbed nerve fibre layer and the absence of regular outer retinal layers. A perimetric examination revealed a relatively superior defect. Magnetic resonance imaging (MRI) revealed the left eye globe showed an abnormal morphology and that the optic nerve was abnormally shaped and shifted nasally in the left eye. Fundus fluorescein angiography (FFA) of the left eye revealed the absence of independent vascular vessels in the disc-like lesion. Hyperfluorescence with patchy fluorescence was evident in the inferotemporal area of the disc. Vascular loops surrounding the temporal region were evident in both eyes. Her right eye was normal except for the vascular loop. We proposed that this represented a case of pseudoduplication of the optic disc. The patient did not undergo any treatment, and her visual acuity remained stable during the follow-up period.

**Conclusions:**

Our patient presented with a deep and ectatic coloboma below the optic disc that communicated with the true optic nerve and was originally thought to indicate a bifurcated optic nerve. This case suggests that atypical ectatic colobomas should be considered before diagnosing malformations related to the optic nerve in double optic disc cases.

## Background

Duplication of the optic disc can be classified as either true duplication or pseudoduplication, both of which are extremely rare clinical conditions [1]. These conditions often confuse paediatric and retinal ophthalmologists because they are hard to distinguish. Pseudoduplication of the optic disc is considered to be generally associated with chorioretinal colobomas [2–7], although a few cases have involved pathological myopia [8], moderate myopia [9], proliferative diabetic retinopathy [10] and even CHARGE syndrome [11]. Typical clinical manifestations of pseudoduplicated optic discs include circumscribed, disc-like lesions with vessels radiating from the defect and bridging vasculature from the central retinal vessels [2–11]. The lesion often arises inferior to the normal disc and consists of a single nerve and apparent cupping surrounded by a ring of chorioretinal atrophy. Clinically, patients generally have decreased visual acuity and superior visual defects without a strong sex or age predominance [2–14].

Here, we report a rare case of pseudodoubling of an optic disc that initially resembled as a bifurcated optic nerve in a strabismus child. This patient, the youngest case of a pseudodoubled disc reported in the literature. She underwent fundus autofluorescence (FAF), fundus fluorescein angiography (FFA), optical coherence tomography (OCT) and magnetic resonance imaging (MRI) examinations, which revealed unusual clinical features that made it difficult to determine whether true duplication or pseudo-doubling of the optic disc had occurred. This study was approved by the Ethical Committee of Xinhua Hospital at the Shanghai Jiao Tong University School of Medicine and conducted in accordance with the principles of the Declaration of Helsinki.

## Case presentation

A 6-year-old female child was initially referred to our hospital due to strabismus and poor vision in the left eye. Upon examination, she was found to have a Snellen’s visual acuity of 8/10 in the right eye with a refraction of + 0.75 dioptres and a visual acuity of 3/100 in the left eye with a refraction of + 1.75/− 1.25 × 175. According to the Hirschberg test, the left eye showed perceptual exotropia of 15 degrees with poor fixation, while the right eye showed exotropia that was 2 degrees higher than that of the left eye. The intraocular pressure was 15 mmHg in the right eye and 13 mmHg in the left eye. The patient was a mature child with a birth weight of 3000 g. There was no history of maternal drug or alcohol abuse during her mother’s pregnancy. There was no family history of ocular problems except for a larger than normal cup-to-disc area ratio in her father’s eyes. The patient’s parents denied a history of trauma or a perforation. The pupils were equal and reactive. The colour vision and anterior segment examination results were bilaterally unremarkable. Fundus imaging of the right eye was normal.

**Fig. 1:**
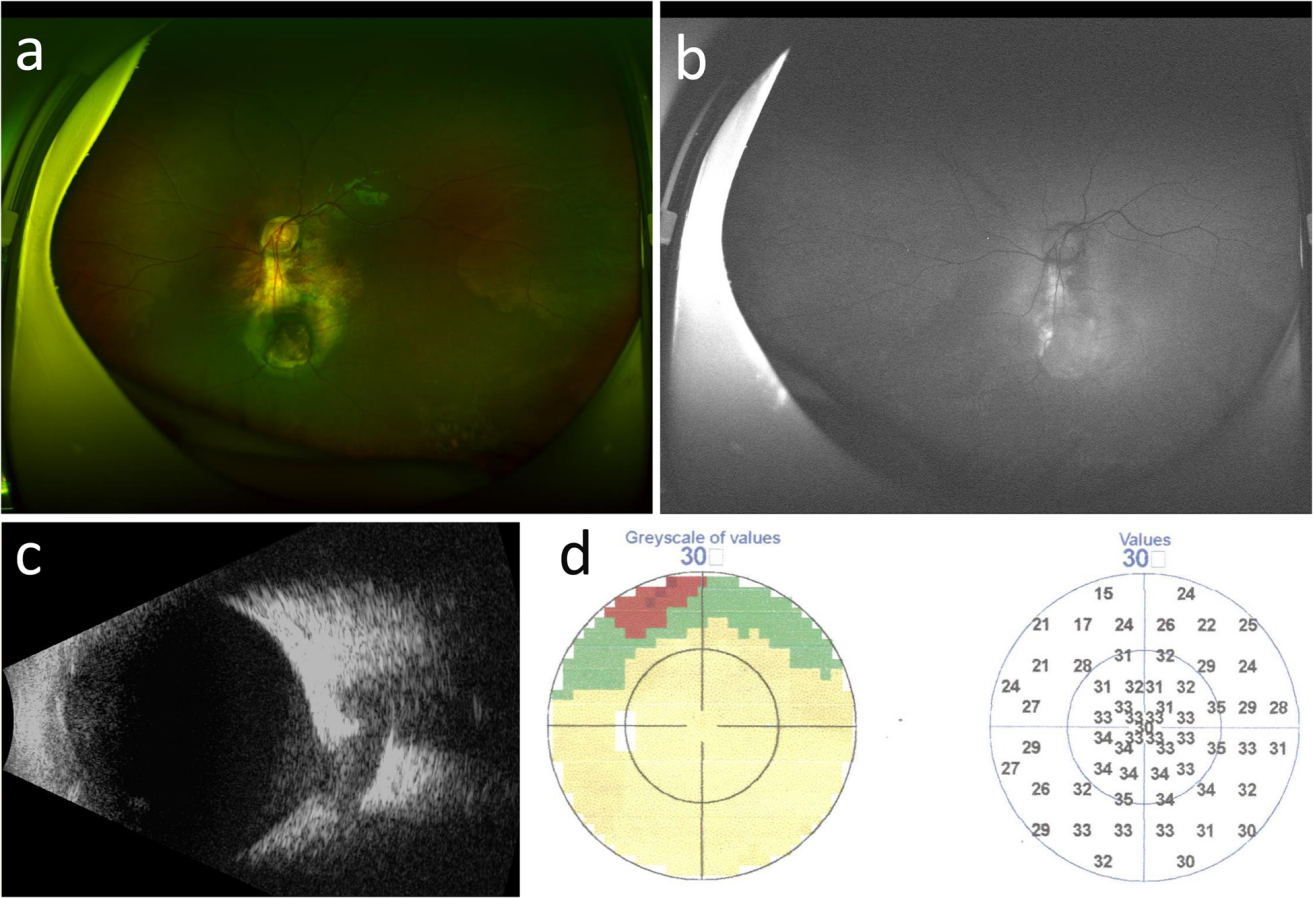
Fundus, FAF, B scan ultrasonography and perimetric examination of the left eye. **a**. Fundus image demonstrating duplication of the optic disc. **b**. FAF image demonstrating that the area located between the true disc and the disc-like lesion showed hyperfluorescence, indicating the accumulation of lipofuscin between the nerve fibre layer and choroidal membrane layer. **c**. B scan ultrasonography presented an ectatic coloboma below the optic disc that communicated with the true optic nerve and therefore resembled a bifurcated optic nerve. **d**. Perimetric examination revealed a relative horizontal superior defect

**Fig. 2:**
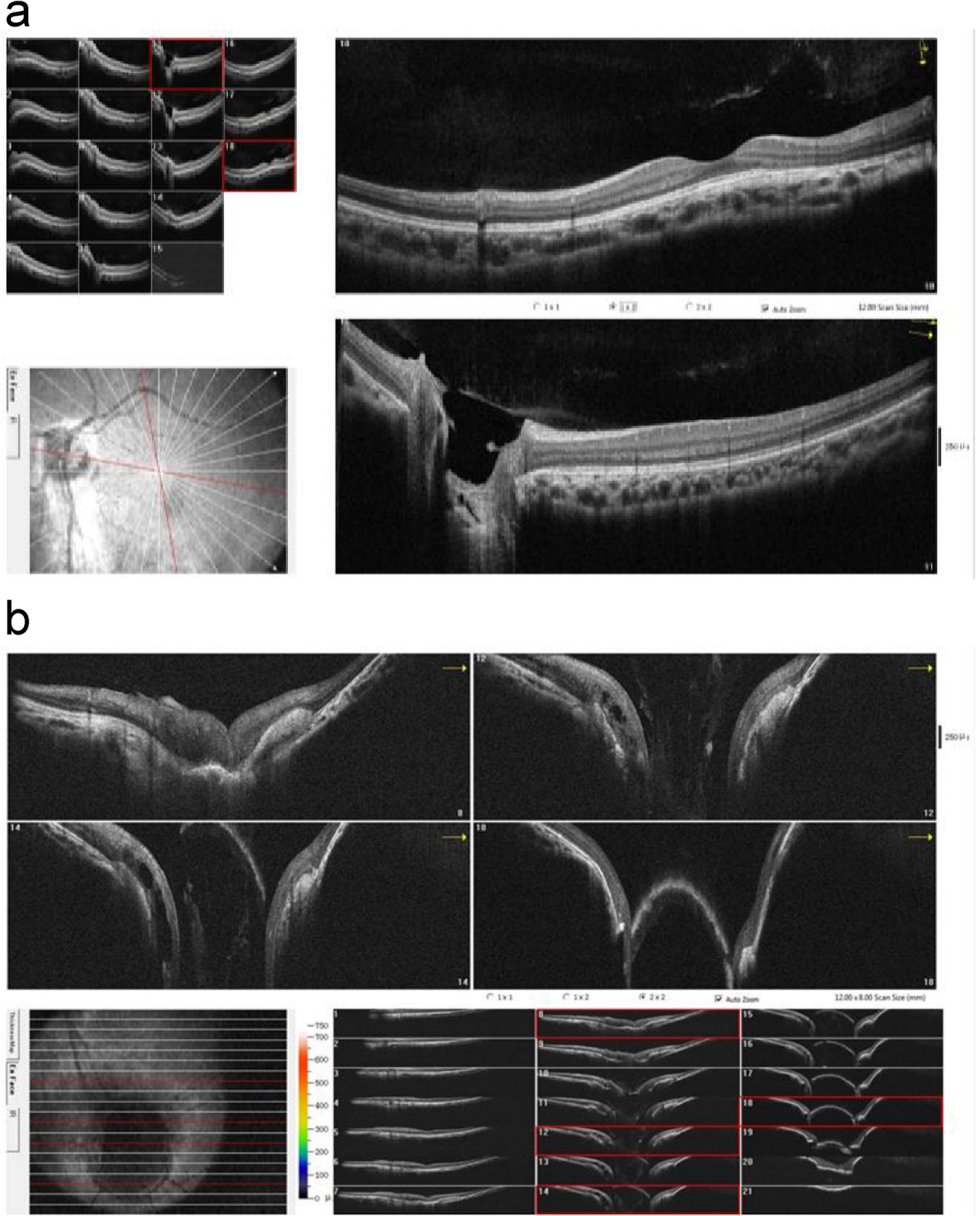
OCT images of the left eye. **a**. OCT examination of the true optic disc was normal. **b**. OCT examination of the disc-like lesions showed a huge and deep crater-like depression in the lesion, a disturbed nerve fibre layer and the absence of regular outer retinal layers

**Fig. 3:**
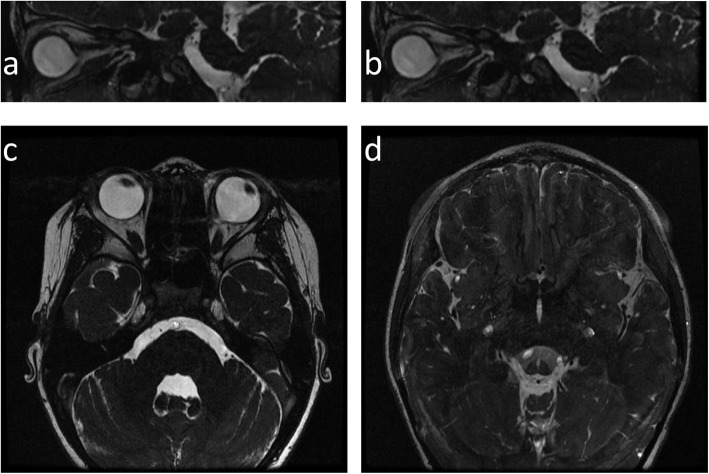
MRI images of the right eye and left eye. **a** and **b**. Sagittal MRI scans of the left eye. **c**. Axial MRI scans of both eyes. **d**. Coronal MRI scans of both eyes. MRI images revealed that the morphology of the globe in the left eye was abnormal and that the abnormally shaped optic nerve in the left eye was partially shifted toward the nasal side. The right eye showed a single optic nerve

However, fundus imaging of the left eye revealed a circumscribed and disc-like lesion located one disc diameter below the true optic disc that showed profound central cupping and resembled a second optic disc with a vascular supply (Fig. 1a). FAF imaging demonstrated that the area located between the true disc and the disc-like lesion was hyperfluorescent, indicating the accumulation of lipofuscin between the nerve fibre layer and choroidal membrane layer (Fig. 1b). Of additional interest were the B scan ultrasonography results of the left eye, which showed an optic nerve with a weak-echo region, indicating that two strands originated from the optic nerve (Fig. 1c). A perimetric examination of the left eye revealed a relatively horizontal superior defect in the nasal region (Fig. 1d). An additional OCT examination showed that the structure of the true optic disc and the macular area of the left eye were normal (Fig. 2a). However, an OCT scan demonstrated a large crater-like depression in the disc-like lesion in the left eye, indicating a colobomatous defect that was covered by a mysterious membranous structure, a disturbed nerve fibre layer and the absence of regular outer retinal layers (Fig. 2b). MRI examination of the orbital region revealed that the morphology of the left eye globe was abnormal and that the abnormally shaped optic nerve in the left eye was shifted partially toward the nasal side. The right eye showed only a single optic nerve (Fig. 3). Initially, it was difficult to determine whether true duplication or pseudoduplication of the optic disc had occurred. Therefore, to more precisely diagnose either true duplication or pseudoduplication of the optic disc, an invasive examination was performed after informed consent was provided by the patient and her guardians.

**Fig. 4:**
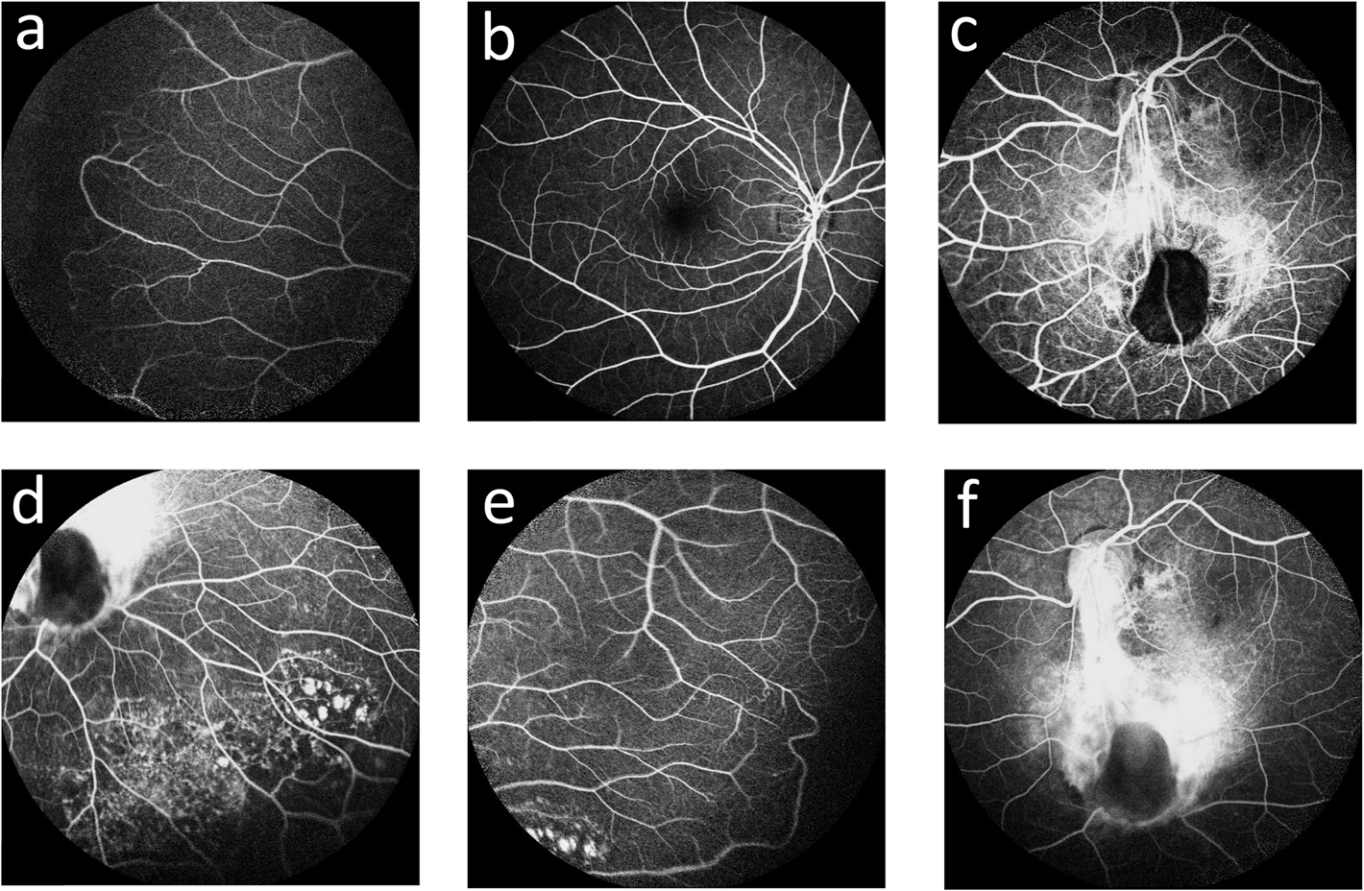
FFA images of the right eye and left eye. **a**. The temporal field of the right eye showed a vascular loop. **b**. The posterior pole of the right eye was normal. **c**. The superior field of the left eye showed a descending venule that drained into the optic disc and divide into 2 branches when it reached the disc-like lesion. Approximately 5 small arterioles and 3 venules departed from the central cupping area. **d**. Hyperfluorescence with patchy fluorescence was evident in the inferotemporal field of the left eye. **e**. The temporal field of the left eye showed a vascular loop. **f**. The inferior disc-like lesions of the left eye showed marked hypofluorescence, and the area located between the optic disc and the lesion showed late hyperfluorescence

According to the FFA examination of the right eye, there was a vascular loop without exudation in the surrounding temporal region (Fig. 4a), and the rest of the region was normal (Fig. 4b). In the left eye, the vessels entering the upper retinal quadrants were normal. A descending venule drained into the optic disc and divided into 2 branches when it reached the disc-like lesion. A vessel from the true disc arched across the disc-like lesion. Approximately 5 small arterioles and 3 venules departed from the central cupping area (Fig. 4c). Hyperfluorescence with patchy fluorescence was evident in the inferotemporal area of the disc (Fig. 4e). A vascular loop without exudation was also found in the surrounding temporal region of the left eye (Fig. 4e). The inferior disc-like lesions showed marked hypofluorescence, and the area located between the optic disc and the lesion showed late hyperfluorescence (Fig. 4f). The fluorescein that filled the arteries of the presumed pseudoduplicate of the optic disc descended from the optic disc instead of from the inferior disc-like lesion. The second disc was also a colobomatous-type disc, but it had an abnormal supply of vessels. The macular area was displaced in the subtemporal region (Fig. 4c and f).

The clinical examination, fundus imaging, perimetric examination, FAF, FFA, OCT, B-scan and MRI findings contributed to the diagnosis of pseudoduplication due to a chorioretinal coloboma that was coincidentally similar in size and shape to a normal optic disc and showed sufficient optic disc involvement to create vascular communication between the excavated second disc and the central retinal vessels. Thus, the ectatic coloboma located below the optic disc communicated with the true optic nerve so that an additional examination gave impression that the optic nerve was bifurcated. The patient did not undergo any treatment, and her visual acuity remained stable during the follow-up period.

## Discussion and conclusions

Both true duplication and pseudoduplication of the optic disc are extremely rare clinical presentations in humans but are common in some teleost fishes in which the optic nerve is divided into several fasciculi [1]. Duplication of the optic disc is equally likely to occur unilaterally or bilaterally. Distinguishing true doubling from pseudodoubling of the optic disc presents a clinical challenge. In the literature, clinical findings, including two discs with separate vascular supplies demonstrated by fluorescein angiography, two optic nerves confirmed by neuroradiological examinations, double blind spots and the presence of nerve fibre layers under the extra disc, must be characterized as typical true doubling [1, 2]. Previous reports have also documented various concurrent clinical findings that can accompany pseudoduplication of the optic discs, including bilateral choroiditis [12] and bilateral optic disc pits [13].

Regarding pseudodoubling of the optic disc, lesions resembling a second optic disc in terms of size, shape and vascularization are often located 0.5–3 disc diameters (DD) inferior to the true disc and show apparent cupping and an associated with surrounding chorioretinal atrophy. Therefore, superior visual field defects are common in affected cases and related to the absence of inferior nerve fibre bundles [3]. Pseudoduplication may cause double blind spots or superior hemifield defects because the position of the disc-like lesion can be superior to the optic disc [6]. OCT scans of the disc-like lesion may reveal a colobomatous defect covered by an undisturbed nerve fibre layer, as was reported in a study by Andonegui J et al. [11]. Thus, double blind spots and the absence of a nerve fibre layer under the disc-like lesion are manifestations observed in both true doubling and pseudo-doubling of an optic disc. A single and normal-shaped optic nerve and bridging retinal vessels that extend between the true optic disc and the disc-like lesion were formerly key features indicating a diagnosis of pseudodoubling [6]. Thus, a careful examination by an ophthalmologist is indispensable to further distinguishing between true doubling and pseudodoubling of optic discs.

In our review of the literature concerning doubling of the optic disc, it became evident that nosological entities such as chorioretinal colobomas must first be considered with regard for the case presented here [2–7]. Failed closure of the foetal fissure results in a typical coloboma during the fourth to fifth weeks of mesoderm development. Pesudovs K and Weisinger HS proposed that their reported case represented a chorioretinal coloboma with optic disc involvement sufficient to create vascular communication between the true disc and the disc-like lesion [13]. The inferior lesion was located in the region of the embryonic fissure [13]. Optic head nerve and chorioretinal colobomas occur due to closure defects in the proximal embryonic fissure at six weeks of gestation and can occur in conjunction; this may be the reason underlying the formation of pseudodoubled discs [2–14].

In our case, the OCT scan demonstrated the absence of a nerve fibre layer and regular retinal structures on both sides of the disc-like lesion. The crater-like depression evident on the OCT scan may have been the result of coloboma and glial tissue proliferation in this case. To the best of our knowledge, our case presented the largest and deepest crater-like depression in an optic disc duplication so far reported in the literature. FFA revealed the absence of an independent vascular supply to the lesion, thus establishing the diagnosis of a pseudo-doubled optic disc. Therefore, we propose that B scan ultrasonography and MRI images revealed an ectatic coloboma below the optic disc that communicated with the true optic nerve and resembled a bifurcated optic nerve.

In addition, FFA examinations are helpful in distinguishing these conditions because a true optic disc will show late hyperfluorescence, whereas a disciform lesion will not [2]. Thus, we propose that the presence of marked hypofluorescence in the lesion could be important evidence indicating a diagnosis of pseudodoubled discs. Although FFA is an effective examination for diagnosing disc pseudo-doubling, colour Doppler also revealed that the disc-like lesion was vascularized by the central retinal artery and the vessels that linked the optic disc [12]. In addition to colour Doppler examinations, there may be another method to verify duplication of the optic disc. In our case, we first identified a hyperfluorescent area between the true disc and disc-like lesion on FAF, indicating the accumulation of lipofuscin between the nerve fibre layer and choroidal membrane layer. This finding may be a characteristic of pseudodoubling of optic discs. More cases are necessary to further evaluate whether this manifestation is helpful in the diagnosis of pseudoduplication of the optic disc.

In conclusion, our case was finally diagnosed as pseudoduplication of the optic disc due to chorioretinal coloboma. Our case exhibited the largest and deepest crater-like depression of a pseudo optic disc among the cases currently reported in the literature. To the best of our knowledge, this is the first case of a pseudodoubled optic disc that presented as a deep and ectatic coloboma below the optic disc that communicated with the true optic nerve and therefore resembled a bifurcated optic nerve. Of additional interest was our finding that this case initially presented as strabismus with a bilateral vascular loop surrounding the temporal region. This indicates that in cases of pseudoduplication of the optic disc, the macular area can be displaced due to abnormal foetal fissure closure; thus, affected patients may initially present with strabismus. Pseudoduplication of the optic disc can also accompany minor problems in the development of surrounding vascular structures. It is difficult to distinguish true doubling from pseudodoubling of the optic disc, and this case suggests that atypical ectatic colobomas should be considered before diagnosing malformations related to the optic nerve in cases presenting a doubled optic disc.

## Data Availability

The datasets used and/or analysed in the current study are available from the corresponding author upon reasonable request.

## Abbreviations

CHARGE: Ocular coloboma (C), heart disease (H), choanal atresia (A), retarded growth and (or) anomalies of the central nervous system (R), genitourinary defects and (or) hypogonadism (G) and ear abnormolities and (or) hearing loss (E)
DD: Disc diameter
FAF: Fundus autofluorescence
FFA: Fundus fluorescein angiography
MRI: Magnetic resonance imaging
OCT: Optical coherence tomography
